# Holstein and Jersey Steers Differ in Rumen Microbiota and Enteric Methane Emissions Even Fed the Same Total Mixed Ration

**DOI:** 10.3389/fmicb.2021.601061

**Published:** 2021-03-18

**Authors:** Mahfuzul Islam, Seon-Ho Kim, Sonny C. Ramos, Lovelia L. Mamuad, A-Rang Son, Zhongtang Yu, Sung-Sil Lee, Yong-Il Cho, Sang-Suk Lee

**Affiliations:** ^1^Ruminant Nutrition and Anaerobe Laboratory, Department of Animal Science and Technology, Sunchon National University, Suncheon, South Korea; ^2^Department of Microbiology and Parasitology, Sher-e-Bangla Agricultural University, Dhaka, Bangladesh; ^3^Department of Animal Sciences, The Ohio State University, Columbus, OH, United States; ^4^Institute of Agriculture and Life Science and University-Centered Labs, Gyeongsang National University, Jinju, South Korea; ^5^Animal Disease and Diagnostic Laboratory, Department of Animal Science and Technology, Sunchon National University, Suncheon, South Korea

**Keywords:** enteric methane emissions, Holstein steer, Jersey steer, rumen fermentation, rumen microbiota

## Abstract

Previous studies have focused on the rumen microbiome and enteric methane (CH_4_) emissions in dairy cows, yet little is known about steers, especially steers of dairy breeds. In the present study, we comparatively examined the rumen microbiota, fermentation characteristics, and CH_4_ emissions from six non-cannulated Holstein (710.33 ± 43.02 kg) and six Jersey (559.67 ± 32.72 kg) steers. The steers were fed the same total mixed ration (TMR) for 30 days. After 25 days of adaptation to the diet, CH_4_ emissions were measured using GreenFeed for three consecutive days, and rumen fluid samples were collected on last day using stomach tubing before feeding (0 h) and 6 h after feeding. CH_4_ production (g/d/animal), CH_4_ yield (g/kg DMI), and CH_4_ intensity (g/kg BW^0.75^) were higher in the Jersey steers than in the Holstein steers. The lowest pH value was recorded at 6 h after feeding. The Jersey steers had lower rumen pH and a higher concentration of ammonia-nitrogen (NH_3_-N). The Jersey steers had a numerically higher molar proportion of acetate than the Holstein steers, but the opposite was true for that of propionate. Metataxonomic analysis of the rumen microbiota showed that the two breeds had similar species richness, Shannon, and inverse Simpson diversity indexes. Principal coordinates analysis showed that the overall rumen microbiota was different between the two breeds. Both breeds were dominated by *Prevotella ruminicola*, and its highest relative abundance was observed 6 h after feeding. The genera *Ethanoligenens*, *Succinivibrio*, and the species *Ethanoligenens harbinense, Succinivibrio dextrinosolvens, Prevotella micans, Prevotella copri, Prevotella oris, Prevotella baroniae*, and *Treponema succinifaciens* were more abundant in Holstein steers while the genera *Capnocytophaga, Lachnoclostridium, Barnesiella, Oscillibacter*, *Galbibacter*, and the species *Capnocytophaga cynodegmi, Galbibacter mesophilus, Barnesiella intestinihominis, Prevotella shahii*, and *Oscillibacter ruminantium* in the Jersey steers. The Jersey steers were dominated by *Methanobrevibacter millerae* while the Holstein steers by *Methanobrevibacter olleyae*. The overall results suggest that sampling hour has little influence on the rumen microbiota; however, breeds of steers can affect the assemblage of the rumen microbiota and different mitigation strategies may be needed to effectively manipulate the rumen microbiota and mitigate enteric CH_4_ emissions from these steers.

## Introduction

Holstein and Jersey are two major dairy breeds for milk production. However, steers of these breeds also contribute to beef production. To meet the ever-increasing demand for milk and beef by the growing global population and rising living standards, improvement of animal performance is required. In the past decade, researchers have embraced next-generation sequencing (NGS) techniques to understand the underlying interactions among diets, the rumen microbiome, and adult ruminants to help improve productivity and reduce the output of wastes (e.g., CH_4_ and NH_3_-N). Recently, O’Hara et al. (2020) reviewed the association of the rumen microbiota and fermentation products and the effects of such association on valuable traits such as feed efficiency and CH_4_ emissions. In the rumen of most ruminant species, *Firmicutes*, *Bacteroidetes*, and *Proteobacteria* are the dominant bacterial phyla, and *Prevotella*, *Fibrobacter*, and *Butyrivibrio* are the dominant bacterial genera, which can ferment a wide range of dietary polysaccharides and protein (O’Hara et al., 2020).

The rumen microbiome contributes either directly or indirectly to animal performance (Islam and Lee, 2018); however, the variation of feed efficiency has a significant association with differences in the rumen microbiome among individual ruminants (Li et al., 2019a; McLoughlin et al., 2020). Therefore, the rumen microbiome can be a potential target to manipulate not only to improve feed efficiency but also to reduce CH_4_ emissions. Enteric CH_4_ is produced primarily through hydrogenotrophic methanogenesis, the reduction of CO_2_ by H_2_ by methanogens (Moss et al., 2000; Ellis et al., 2008). CH_4_ is not only a potent greenhouse gas (GHG) but also represents a gross energy loss (Johnson and Johnson, 1995; Appuhamy et al., 2016). Earlier studies on CH_4_ emissions found inconsistent results between Holstein and Jersey dairy cows, with much less information available for steers of both breeds. Münger and Kreuzer (2006, 2008) reported that CH_4_ yield (g/kg dry matter intake; DMI) was not different between Holstein and Jersey cows. In contrast, Olijhoek et al. (2018) reported a significantly higher CH_4_ yield in Jersey cows than in Holstein cows, either fed high-forage or high-concentrated diets or either allowed for a low or high feed refusal. *Methanobrevibacter* is the dominant genus of methanogens and the major CH_4_ producer in ruminants (Leahy et al., 2013; Henderson et al., 2015). King et al. (2011) reported that the *Methanobrevibacter smithii, Methanobrevibacter gottschalkii, Methanobrevibacter millerae*, and *Methanobrevibacter thaueri* (collectively known as SGMT) group of *Methanobrevibacter* were more abundant in Jersey cows than in Holstein cows, and it had a positive correlation with CH_4_ production. However, Cersosimo et al. (2016) reported similar communities of methanogens between Holstein and Jersey cows.

Diet plays a significant role in shaping the rumen microbiome, leading to differences in the rumen fermentation characteristics and CH_4_ emissions (Spor et al., 2011). Notably, types of diet such as high-forage vs. high-concentrate diets had a profound influence on the rumen microbiome, its fermentation characteristics, and enteric CH_4_ production (Zhang et al., 2017; Li et al., 2019b; Wang et al., 2020). Hence, to minimize the dietary influence on rumen microbiome when comparing different breeds, identical feed should be fed. As reported by Li et al. (2019a), host genetics has some influence on rumen microbiome and feed efficiency in several beef breeds, including Angus, Charolais, and Kinsella composite hybrid, but the effect on CH_4_ emissions remains undetermined. Another study showed that both rumen microbiome and host genetics could have a joint association with CH_4_ emissions in lactating Holstein cows (Difford et al., 2018), but no data on rumen fermentation parameters were reported. Several previous studies showed differences in rumen microbiome between Holstein and Jersey cows. For instance, Beecher et al. (2014) reported that the relative abundance of *Ruminococcus flavefaciens* was higher in Holstein cows than in Jersey cows, whereas no difference was observed in *Fibrobacter succinogenes*. Paz et al. (2016) reported that lactating Holstein and Jersey cows harbored a distinct rumen bacterial microbiota, with the former having a greater diversity and species richness than the latter. *Prevotellaceae* is the largest bacterial family in both Holstein and Jersey cows, but *Lachnospiraceae* and candidate family p-2534-18B5 were more abundant in Holstein cows than in Jersey cows (Paz et al., 2016). Despite the numerous previous studies on the rumen microbiome of Holstein and Jersey dairy cows, there is limited information on the rumen microbiome in growing steers, especially steers of dairy breeds. To bridge this knowledge gap, comparative studies are needed. We hypothesized that Holstein and Jersey steers might harbor different rumen microbiome, which might influence their rumen fermentation and enteric CH_4_ emissions. The objective of the present study was to test the above hypothesis by comparing the rumen microbiota, rumen fermentation characteristics, and methane emissions.

## Materials and Methods

### Animal, Experimental Design, and Diet

The animal experiment was conducted at the Sunchon National University (SCNU) Animal Farm under normal environmental conditions with a temperature-humidity index of 65.67 ± 2.22. The laboratory analysis was performed at the Ruminant Nutrition and Anaerobe Laboratory, Department of Animal Science and Technology, SCNU, Jeonnam, South Korea.

Six Holstein (710.33 ± 43.02 kg) and six Jersey (559.67 ± 32.72 kg) steers, both non-cannulated, at the age of about 27 months were fed the same total mixed ration (TMR) for 30 days, with the first 25 days for diet adaptation and the remaining 5 days for determination of DMI (the whole 5 days), CH_4_ measurement (the first 3 days), and rumen fluid sample collection (the last day). All steers were kept in individual stalls with feeding and water facilities. The steers were offered the same TMR (Table 1) once a day at 0900 h at a rate of 5–10% of refusal. DMI was measured as the difference between feed offered and the feed refusal. The average body weight (BW) was calculated as (the initial BW + the final BW)/2, and average daily gain (ADG) was recorded for the last 10 days of the experimental period. The TMR was sampled twice (at days 7 and 28) during the feeding trial, and the dry matter content was determined using a hot-air oven at 65°C for 72 h (Bharanidharan et al., 2018). The chemical composition of the TMR was analyzed following the standard methods (Aoac, 2005). The content of neutral detergent fiber (NDF) and acid detergent fiber (ADF) was determined per the protocols described by Van Soest (1973) and Van Soest et al. (1991), respectively.

**TABLE 1:** Ingredients and chemical composition of the TMR fed to the steers.

Ingredients	Compositions (% of DM)
Corn grain	36.80
Corn gluten feed	17.89
Lupin	12.49
Wheat bran	11.61
Oat hay	20.26
Limestone (1 mm size)	0.68
Vitamin premix^1^	0.07
Mineral premix^2^	0.07
Salt	0.14
Total	100.00
**Chemical composition (% as DM basis)**	
DM (% as fed basis)	73.06
Crude protein	19.86
Crude fiber	9.23
Crude fat	4.60
Ash	7.56
Calcium	0.74
Phosphorous	0.40
NDF	32.17
ADF	14.29

*^1^The vitamin premix contained (g/kg) L-ascorbic acid, 121.2; DL-α-tocopherol acetate, 18.8; thiamin hydrochloride, 2.7; riboflavin, 9.1; pyridoxine hydrochloride, 1.8; niacin, 36.4; Ca-D-pantothenate, 12.7; myo-inositol, 181.8; D-biotin, 0.27; folic acid, 0.68; p-aminobenzoic acid, 18.2; menadione, 1.8; retinal acetate, 0.73; cholecalciferol, 0.003; and cyanocobalamin, 0.003; and the remaining was cellulose. ^2^The mineral premix contained (g/kg) MgSO_4_ ⋅ 7H_2_O, 80.0; NaH_2_PO_4_ ⋅ 2H_2_O, 370.0; KCl, 130.0; ferric citrate, 40.0; ZnSO_4_ ⋅ 7H_2_O, 20.0; Ca-lactate, 356.5; CuCl_2_, 0.2; AlCl_3_ ⋅ 6H_2_O, 0.15; KI, 0.15; Na_2_Se_2_O_3_, 0.01; MnSO_4_ ⋅ H_2_O, 2.0; and CoCl_2_ ⋅ 6H_2_O, 1.0. DM, dry matter; NDF, neutral detergent fiber; ADF, acid detergent fiber.*

### Enteric CH_4_ Measurements

Enteric CH_4_ emissions were measured using a GreenFeed unit (C-Lock Inc., Rapid City, SD, United States) as described by Hammond et al. (2015) and Hristov et al. (2015) with minor modifications. Briefly, CH_4_ emissions were measured for each steer at eight different time points (00:00, 03:00, 06:00, 09:00, 12:00, 15:00, 18:00, and 21:00) for three consecutive days during the measurement period. The GreenFeed unit was installed in one corner of a large pan. At each measurement time, all steers were transferred to that pan from their stalls one after another. Each steer was allowed for access to the GreenFeed unit for approximately 10 min. Molasses-coated concentrated pellets were used to attract the animals to the GreenFeed unit and ensure a proper head-down position within the hood for the measuring duration. The amount of pellets ingested by each steer during the measuring duration was not included in the DMI calculation. The entry and exit times for each animal, standard gas calibration, and CO_2_ recovery data were recorded and sent to C-Lock Inc., which calculated the CH_4_ emissions. The calculated data were received via a web-based data management system, and CH_4_ emissions were expressed as CH_4_ production (g/d), CH_4_ yield (g/kg DMI), and CH_4_ intensity (g/kg BW^0.75^).

### Sample Collection and Processing

Rumen fluid samples were collected from each of the steers at two different time points: before feeding (0 h) and 6 h after feeding using stomach tubing on the last day of the experiment. To minimize contamination from the saliva, the first 300 ml of each rumen fluid sample was discarded. The pH was measured immediately after collection with a pH meter (Seven Compact^TM^ pH/Ion meter S220, Mettler Toledo, Switzerland). The rumen fluid sample collected from each steer was divided into three separate aliquots, which were transferred to the laboratory and stored at −80°C until subsequent analysis for ammonia nitrogen (NH_3_-N), volatile fatty acid (VFA), and rumen microbiota.

### Analysis of NH_3_-N and VFA Concentrations

The concentration of NH_3_-N was measured colorimetrically using a Libra S22 spectrophotometer (CB40FJ; Biochrom Ltd., Cambourne, United Kingdom) following the protocol described by Chaney and Marbach (1962). The VFA concentration was measured according to the methods described by Tabaru et al. (1988) and Han et al. (2005) using high-performance liquid chromatography (HPLC; Agilent Technologies 1200 series, Waldbronn, Baden-Wurttemberg, Germany). A UV detector (set at 210 and 220 nm), a METACARB87H column (Varian, Palo Alto, CA, United States), and a buffered solvent (0.0085 N H_2_SO_4_) at a flow rate of 0.6 mL/min were used to run the HPLC.

### DNA Extraction and Metataxonomic Analysis

The rumen fluid samples (two per each of the 12 steers, 24 in total) were sent to Macrogen Inc. (Seoul, South Korea) for DNA extraction and metataxonomic analysis of the rumen microbiota. Briefly, the DNA was extracted using a PowerSoil^®^ DNA Isolation Kit (Cat. No. 12888, MO BIO) following the manufacturer’s protocol (Claassen et al., 2013). The quality and quantity of DNA were checked using PicoGreen and Nanodrop. The Illumina 16S Metagenomic Sequencing Library protocols were followed to prepare the amplicon library of each sample, which uses two-step PCR amplification of the V3–V4 region of the 16S rRNA genes using primers Bakt_341F (5′-AGATGTGTATAAGAGACAG-3′) and Bakt_805R (5′-GATGTGTATAAGAGACAGG-3′) (Klindworth et al., 2013), with multiplexing indices and Illumina sequencing adapters being introduced in the second PCR (10 cycles). The individual amplicon libraries were normalized after quantification using PicoGreen, sizes-verified using the TapeStation DNA ScreenTape D1000 (Agilent), pooled at an equal molar ratio, and then sequenced on a MiSeq system (Illumina, San Diego, CA, United States) using the 2 × 300 bp kit. The raw sequence data were trimmed by Trimmomatic (v0.38) (Bolger et al., 2014), and the two reads were joined using the FLASH (1.2.11) program (Magoč and Salzberg, 2011). Sequences shorter than 400 bp were discarded. The rDnaTools was used to identify and remove chimeric sequences^1^. Samples were subsampled to an even depth of 10,000 sequences per sample to avoid bias generated due to different sequencing depths. The quality-filtered sequences were clustered into operational taxonomic units (OTU) at 97% sequence similarity using CD-HIT-OTU (Li et al., 2012). The representative sequence of each OTU was compared using BLASTN (v2.4.0) (Zhang et al., 2000) against the 16S Microbial DB of NCBI^2^ for taxonomic assignment. Alpha diversity measurements including the Shannon diversity index, Inverse Simpson diversity index, and Chao1 richness estimate were determined using QIIME (v1.8). The overall rumen microbiota was compared using principal coordinates analysis (PCoA) based on unweighted UniFrac distance. In addition, the Bray–Curtis distance dissimilarity matrix was used to produce PCoA ellipse at 95% confidence using R (v3.6.2 and package vegan 2.5-6) to assess the difference in overall rumen microbiota between the two breeds.

### Statistical Analysis

All the data of growth performance and CH_4_ emissions were analyzed using the independent group *t*-test (PROC TTEST), and the data of rumen fermentation, alpha diversity measurements and relative abundance of individual taxa of the rumen microbiota were analyzed using the Mixed procedure in SAS (version 9.4; SAS Institute Inc., Cary, NC, United States) (SAS, 2013). The model included the fixed effects of breed, sampling hour, and the interaction between breeds and sampling hours, and the random effect included the steers nested within the breeds. Significant difference was declared when *P*-value was <0.05, while trend was declared at 0.05 < *P* ≤ 0.1.

## Results

### Growth Performance and Enteric CH_4_ Emissions

The growth performance of and CH_4_ emissions from the Holstein and the Jersey steers were shown in Table 2. The Holstein steers had a significantly higher average BW and metabolic BW (BW^0.75^) than the Jersey steers (*P* < 0.05). Similarly, the Holstein steers had a higher (*P* < 0.05) DMI (kg/d) and ADG (kg/d) than the Jersey steers (17.49 vs. 13.57 and 1.48 vs. 1.02, respectively). However, DMI (kg/100 kg BW) was similar in both breeds. The CH_4_ production (g/d), CH_4_ yield (g/kg DMI), and CH_4_ intensity (g/kg BW^0.75^) were higher in the Jersey steers than in the Holstein steers (226.49 vs. 165.46, 16.89 vs. 9.69, and 1.96 vs. 1.19, respectively; *P*-value: 0.124, 0.041, and 0.054, respectively).

**TABLE 2:** Dry matter intake, average daily gain, and methane emissions of Holstein and Jersey steers.

Parameters	Holstein	Jersey	SEM	*P*-value
Average BW (kg)	743.72	582.53	15.506	<0.001
Average BW^ 0.75^ (kg)	142.37	118.55	2.276	<0.001
DMI (kg/d)	17.49	13.57	0.221	<0.001
DMI (kg/100 kg BW)	2.35	2.33	0.026	0.533
DMI (g/kg BW^0.75^)	122.86	114.47	0.708	<0.001
ADG (kg)	1.48	1.02	0.078	0.021
CH_4_ production (g/d)	165.46	226.49	22.222	0.124
CH_4_ yield (g/kg DMI)	9.69	16.89	1.691	0.041
CH_4_ intensity (g/kg BW^0.75^)	1.19	1.96	0.197	0.054

*BW, body weight; DMI, dry matter intake; ADG, average daily gain; CH_4_, methane; SEM, standard error of the mean.*

### Rumen Fermentation Characteristics

The rumen fermentation parameters of the Holstein and Jersey steers at the two sampling different time points were analyzed and the results are presented in Table 3. A lower rumen pH (by about 0.2 pH unit) was recorded in the Jersey steers than in the Holstein steers at both sampling hours (*P* = 0.013), and the pH tended to be lower at 6 h than at 0 h in both breeds (*P* = 0.071). The NH_3_-N concentration (mg/dl) was significantly higher (*P <* 0.05) in the Jersey steers (5.14 and 5.72 at 0 h and 6 h, respectively) than in the Holstein steers (2.98 and 2.94 at 0 h and 6 h, respectively), but it was not affected by sampling hour (*P* > 0.05). The total VFA concentration and the molar proportion of acetate were numerically higher in the Jersey steers, while the molar proportion of propionate was numerically higher in the Holstein steers (*P* > 0.05). The butyrate concentration was significantly increased over time after feeding in both breeds (*P* < 0.05), while acetate and propionate concentrations were numerically increased over time in both breeds (*P* > 0.05).

**TABLE 3:** Rumen fermentation characteristics of Holstein and Jersey steers.

Parameters	Holstein		Jersey		*SEM*	*P*-value		
	0 h	6 h	0 h	6 h		Breed	Hour	B × H
pH	6.84	6.70	6.63	6.46	0.080	0.013	0.071	0.896
NH_3_-N (mg/dl)	2.98	2.94	5.14	5.72	0.645	0.002	0.698	0.658
Total VFA (mM)	68.46	74.89	66.08	77.01	5.028	0.980	0.110	0.660
Acetate (mM)	43.40	46.47	41.79	48.61	3.683	0.945	0.218	0.630
Propionate (mM)	13.08	15.12	12.09	14.50	1.317	0.636	0.206	0.914
Butyrate (mM)	11.99	13.30	12.20	13.90	0.302	0.196	0.001	0.527
Acetate (%)	62.80	62.13	62.99	63.20	1.597	0.713	0.895	0.797
Propionate (%)	19.34	19.99	18.40	18.68	1.300	0.464	0.760	0.900
Butyrate (%)	17.86	17.87	18.61	18.13	0.977	0.633	0.822	0.812
A: P ratio	3.28	3.18	3.46	3.44	0.301	0.486	0.841	0.909

*NH_3_-N, ammonia-nitrogen; VFA, volatile fatty acids; A: P, acetate: propionate ratio; SEM, standard error of the mean.*

### Species Richness, Diversity, and Composition of Rumen Microbiota

**FIGURE 1:**
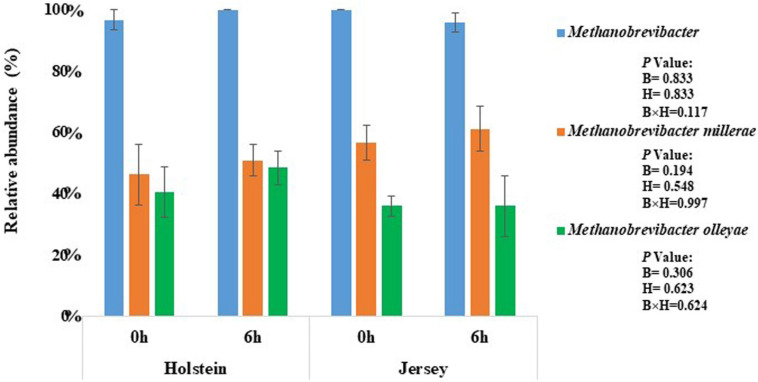
Methanogens identified in the rumen of the Holstein and the Jersey Steers. Blue bars, *Methanobrevibacter*; orange bars. *M. millerae*; and green bars, *M. olleyae*.

A total of 456,397 quality-filtered sequences resulted from processing more than 3 million raw reads produced from sequencing the 24 rumen fluid samples. On average more than 16,800 sequences were obtained for each sample, and the Good’s coverage reached at least 99% (Table 4). The two breeds had a similar number of observed OTUs, Chao 1 richness estimate, Shannon, and Simpson diversity indexes (*P* > 0.05). At the domain level, bacteria (99.78–99.86%) were dominant among the rumen microbiota followed by archaea (0.14–0.22%). The two breeds did not differ in the relative abundance of either domain irrespective of sampling hour (*P* > 0.05) (Supplementary Table 1). Among the archaea, *Methanobrevibacter* of the phylum *Euryarchaeota* was the most predominant genus in both breeds and at both sampling hours (*P* > 0.05) (Figure 1 and Supplementary Table 1). *M. millerae* and *M. olleyae* were the dominant species among the methanogens in both breeds and at both sampling hours; however, *M. millerae* was numerically more predominant in the Jersey steers while *M. olleyae* was more predominant in the Holstein steers (*P* > 0.05) (Figure 1 and Supplementary Table 1).

**TABLE 4:** Sequence reads and alpha diversity measurements of rumen microbiota of Holstein and Jersey steers.

Parameters	Holstein		Jersey		*SEM*	*P*-value		
	0 h	6 h	0 h	6 h		Breed	Hour	B × H
Sequence reads	19,953	20,308	18,980	16,825	1507	0.179	0.580	0.442
OTUs observed	752.67	733.17	736.67	692.17	20.07	0.186	0.140	0.555
Chao 1 estimate	866.75	846.39	858.96	833.67	21.24	0.653	0.322	0.914
Shannon diversity index	7.36	7.31	7.41	7.35	0.091	0.635	0.537	0.936
Inverse Simpson diversity index	0.98	0.98	0.99	0.99	0.002	0.257	0.701	0.701
Goods coverage (%)	99.3	99.3	99.2	99.0	0.100	0.067	0.344	0.271

*OTUs, operational taxonomic units; SEM, standard error of the mean.*

**FIGURE 2:**
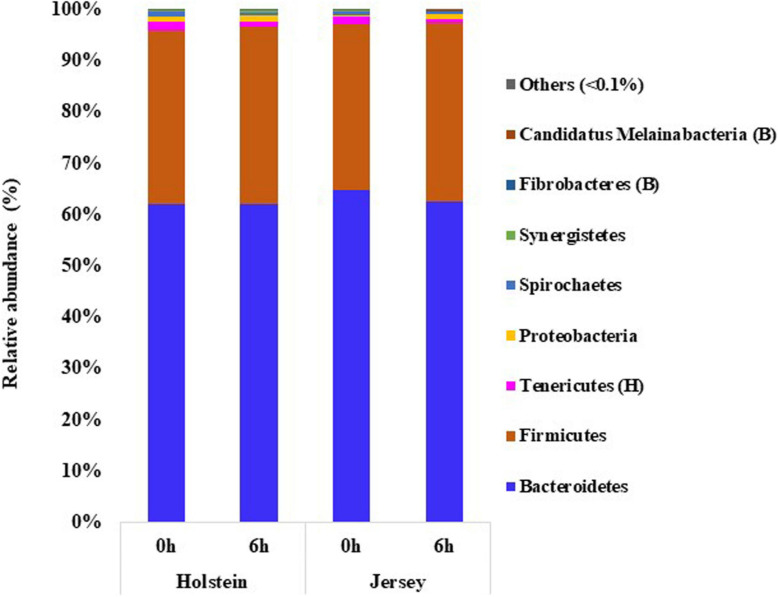
Phyla of bacteria identified in the rumen of the Holstein and the Jersey steers. B, H, and B × H indicate significant (*P* < 0.05) difference in relative abundance between breeds, sampling hours, and the interaction between breed and sampling hours, respectively.

*Bacteroidetes* (61.98–64.84%) followed by *Firmicutes* (32.17–34.76%) were the largest bacterial phyla in both breeds and at both sampling hours, together representing >95% of all bacteria, and these two phyla did not differ (*P* > 0.05) between the two breeds (Figure 2 and Supplementary Table 1). *Tenericutes* and *Proteobacteria* were the next two predominant phyla each with a relative abundance >0.1%, which were also not influenced by breed (*P* > 0.05). The relative abundance of *Tenericutes*, however, was lower at 6 h than at 0 h after feeding (*P* < 0.05). *Fibrobacteres* and Candidatus *Melainabacteria* were the least abundant phyla, and they differed significantly (*P* < 0.05) between the two breeds, with *Fibrobacteres* being more abundant in the Holstein steers, while Candidatus *Melainabacteria* being more abundant in the Jersey steers.

**FIGURE 3:**
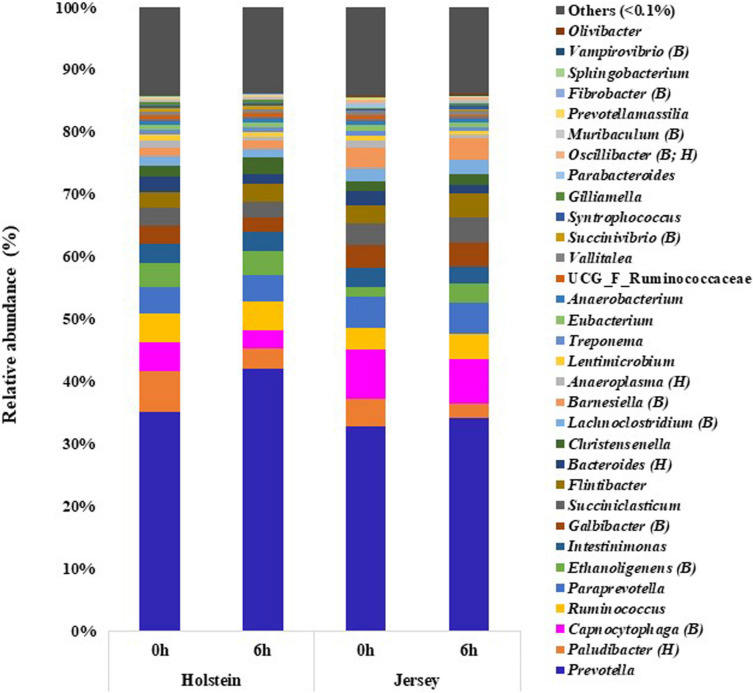
Major genera (each having relative abundance ≥0.1%) of bacteria identified in the rumen of the Holstein and the Jersey steers. B and H indicate significant (*P* < 0.05) differences in relative abundance between breeds and sampling hours, respectively.

Thirty-two of the 212 identified bacterial genera had a relative abundance ≥0.1% at least in one breed and at one of the two sampling hours (Figure 3 and Supplementary Table 2). *Prevotella* was the most abundant bacterial genus regardless of breed or sampling hour, and it tended (*P* = 0.068) to be more predominant in the Holstein steers (35.19 and 42.11%, at 0 and 6 h, respectively) than in the Jersey steers (32.79 and 34.17%). The relative abundance of the genera *Ethanoligenens*, *Succinivibrio*, and *Muribaculum* was significantly higher in the Holstein steers, whereas *Capnocytophaga*, *Galbibacter*, *Lachnoclostridium*, *Barnesiella*, and *Oscillibacter* were more predominant in the Jersey steers (*P <* 0.05). *Paludibacter, Ruminococcus, Paraprevotella, Intestinimonas, Succiniclasticum, Flintibacter, Bacteroides*, and *Christensenella* were the other abundant bacterial genera (each with a relative abundance >1%) in both breeds and at both sampling hours, although there were no significant differences between the two breeds (*P >* 0.05). Sampling time did not influence genus-level relative abundance (*P >* 0.05) except for that of *Paludibacter, Bacteroides, Anaeroplasma*, and *Oscillibacter*, which had a higher relative abundance at 0 h than at 6 h (*P <* 0.05).

**FIGURE 4:**
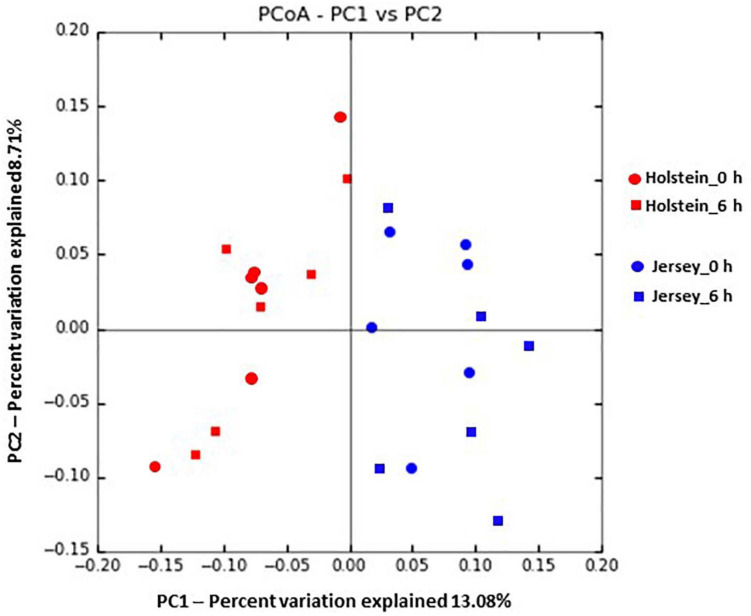
Principal coordinates analysis plot based on unweighted-Unifrac distance showing the comparison of the overall rumen microbiota of the Holstein and the Jersey steers.

**FIGURE 5:**
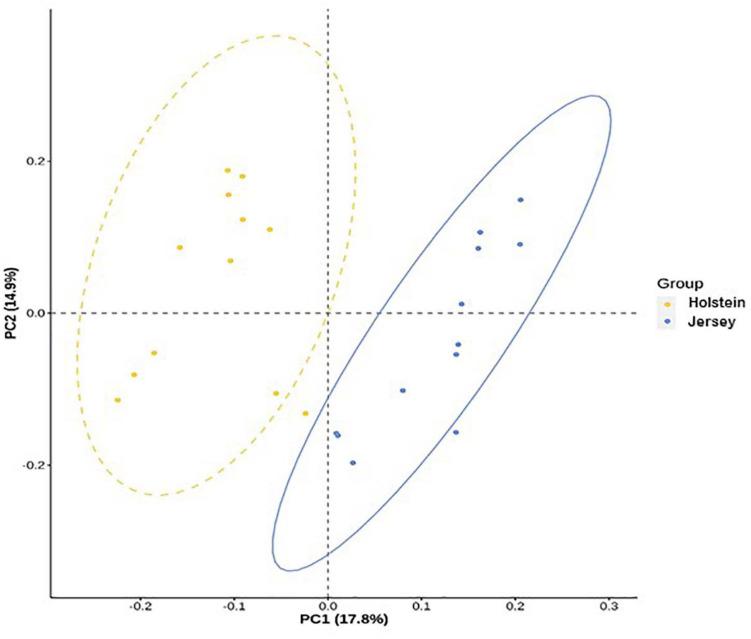
Principal coordinates analysis plot based on Bray–Curtis dissimilarity with ellipse at 95% confidence showing the comparison of the overall rumen microbiota of the Holstein and the Jersey steers.

In total, 377 species or species-level OTUs of bacteria were identified across all the rumen samples, and 20 of them each had a relative abundance ≥1% at least in one breed and at one sampling hour (Table 5). Also, 7 species each had a relative abundance ≥0.5% at least in one breed and at one sampling hour, and they showed significant differences between the two breeds (Table 5). *Pr. ruminicola* was the most abundant bacterial species, and it was not affected by breeds (*P* > 0.05), but it tended to be more predominant at 6 h than at 0 h (*P* = 0.069). Similarly, *Paludibacter propionicigenes, Paraprevotella clara, Ruminococcus bromii, Prevotella brevis, Intestinimonas butyriciproducens, Succiniclasticum ruminis, Flintibacter butyricus, Bacteroides clarus, Prevotella oralis*, and *Christensenella massiliensis* (each has relative abundance ≥1%) did not differ between the two breeds (*P* > 0.05). *Ethanoligenens harbinense, Prevotella micans, Prevotella copri, Prevotella oris, Millionella massiliensis, Succinivibrio dextrinosolvens, Prevotella baroniae, Acetivibrio alkalicellulosi*, and *Treponema succinifaciens* were significantly more abundant in the Holstein steers, whereas *Capnocytophaga cynodegmi, Galbibacter mesophilus, Barnesiella intestinihominis, Clostridium asparagiforme, Prevotella shahii, O. ruminantium, Erysipelothrix larvae*, were significantly more abundant in the Jersey steers (*P* < 0.05). Based on PCoA and PCoA ellipse, the overall rumen microbiota was different between the Holstein and the Jersey steers (Figures 4, 5) even fed with the same TMR; however, no separation of overall rumen microbiome by sampling hour was noted on the PCoA plot (Figure 4).

**TABLE 5:** Major species of bacteria (each having relative abundance ≥1%) and the species that both had relative abundance ≥0.5% at least in one breed and at one sampling hour and differed between the two breeds.

Phylum	Species	Holstein		Jersey		SEM	*P*-value		
		0 h	6 h	0 h	6 h		Breed	Hour	B × H
*Bacteroidetes*	*Prevotella ruminicola*	22.61	29.19	22.52	24.49	2.153	0.292	0.069	0.312
	*Paludibacter propionicigenes*	6.40	3.11	4.41	2.36	0.922	0.201	**0.018**	0.553
	*Paraprevotella clara*	4.22	4.18	4.96	5.00	1.361	0.609	0.999	0.980
	*Capnocytophaga cynodegmi*	3.90	2.48	7.13	6.52	0.708	**<0.001**	0.198	0.596
	*Prevotella brevis*	3.01	3.13	2.42	1.92	0.491	0.114	0.738	0.571
	*Galbibacter mesophilus*	2.95	2.30	3.65	3.69	0.406	**0.024**	0.485	0.433
	*Prevotella micans*	1.53	1.36	0.71	0.63	0.247	**0.010**	0.659	0.859
	*Bacteroides clarus*	1.51	0.75	1.15	0.41	0.357	0.410	0.085	0.979
	*Prevotella oralis*	1.43	1.79	1.98	2.40	0.449	0.237	0.417	0.946
	*Barnesiella intestinihominis*	1.33	1.40	3.00	3.39	0.385	**<0.001**	0.574	0.698
	*Prevotella copri*	1.14	0.94	0.61	0.54	0.186	**0.036**	0.542	0.757
	*Prevotella oris*	0.77	1.00	0.41	0.55	0.130	**0.014**	0.224	0.769
	*Prevotella shahii*	0.52	0.40	0.79	1.00	0.119	**0.003**	0.719	0.201
	*Millionella massiliensis*	0.72	0.72	0.49	0.49	0.107	**0.043**	0.976	1.000
	*Prevotella baroniae*	0.57	0.87	0.13	0.09	0.153	**0.004**	0.488	0.358
*Firmicutes*	*Ethanoligenens harbinense*	4.01	3.99	1.68	3.03	0.529	**0.010**	0.264	0.249
	*Ruminococcus bromii*	3.35	3.65	2.24	3.12	0.468	0.126	0.264	0.576
	*Intestinimonas butyriciproducens*	3.00	2.99	2.95	2.77	0.528	0.798	0.859	0.873
	*Succiniclasticum ruminis*	2.78	2.53	3.48	4.04	0.719	0.152	0.838	0.585
	*Flintibacter butyricus*	2.56	2.93	2.95	3.87	0.382	0.104	0.115	0.493
	*Christensenella massiliensis*	1.33	2.11	1.18	1.33	0.330	0.192	0.193	0.374
	*Clostridium asparagiforme*	1.22	0.97	1.83	1.87	0.244	**0.006**	0.682	0.557
	*Acetivibrio alkalicellulosi*	0.55	0.40	0.22	0.18	0.091	**0.018**	0.396	0.585
	*Oscillibacter ruminantium*	0.25	0.18	0.64	0.50	0.042	**<0.001**	**0.032**	0.472
	*Erysipelothrix larvae*	0.17	0.16	0.50	0.33	0.083	**0.033**	0.411	0.464
*Proteobacteria*	*Succinivibrio dextrinosolvens*	0.58	0.53	0.05	0.25	0.149	**0.039**	0.680	0.502
*Spirochaetes*	*Treponema succinifaciens*	0.54	0.41	0.14	0.11	0.081	**0.003**	0.458	0.606

*SEM, Standard error of the mean. P-values ≤ 0.05 were bolded, while those >0.05 but ≤0.1 were underlined.*

## Discussion

The findings of the present study showed that the Holstein steers had a greater DMI (by 3.92 kg) and ADG (by 0.46 kg/d) than Jersey steers when fed the same TMR diet. This is consistent with the heavier BW and BW^0.75^ of the Holstein steers than the Jersey steers and the report by Flay et al. (2019). The higher DMI by Holstein compared to the Jersey lactating and dry cows was also observed in previous studies (Prendiville et al., 2011; Beecher et al., 2014). However, Prendiville et al. (2011) also reported similar intake capacity (kg DM/100 kg BW) between these two breeds during dry period (1.95 vs. 2.04 in Holstein and Jersey, respectively). Likewise, our study observed non-significant differences of DMI (kg/100 kg BW) between breeds (2.35 vs. 2.33 in Holstein and Jersey, respectively). This finding as well as the same aged steer, used for feeding trial, minimizes the dietary influence of this study. Ruminal pH decreases with the increase of VFAs production by microbial fermentation or subsequently less absorption via the ruminal epithelium, and the less amount of saliva, which acts as a buffering agent, entered the rumen (Aikman et al., 2011). In the present study, the decreasing trend of pH over the sampling time might be due to the numerically increased production of VFAs over time. However, the significantly lower rumen pH observed in the Jersey steers compared to the Holstein steers might be due to the variation in the ruminal absorption and/or production of saliva between the two breeds. On the other hand, according to the Henderson–Hasselbalch equilibrium of pH, [pH = p*K*_*a*_ + log {(Acid^–^)/(HAcid)}; where p*K*_*a*_ is the negative log of the acid constant *K*_*a*_], pH reduction potential of acetic acid (p*K*_*a*_ 4.76) is comparatively higher followed by butyric acid (p*K*_*a*_ 4.82) and propionic acid (p*K*_*a*_ 4.87) (Dijkstra et al., 2012). Therefore, the significantly lower pH in the Jersey steer further explained with the higher proportion of acetate and butyrate compared to the Holstein steers which had higher propionate proportion in this study. Rumen NH_3_-N concentration can be influenced by dietary protein breakdown, NH_3_ utilization by microbes, absorption by rumen wall, and urea hydrolysis in the rumen. The higher rumen NH_3_-N concentration noted in the Jersey steers probably indicates higher breakdown of dietary protein and/or less utilization by the rumen microbes or less ruminal absorption in the Jersey steers. The production of VFA is proportional to DMI, whereas the VFA concentrations in the rumen depend on the differences between the amount of production and the rate of absorption by the ruminal epithelium (Dieho et al., 2016). An earlier study reported that the Holstein dairy cows had a significantly higher molar proportion of propionate and lower proportion of acetate than the Jersey dairy cows (Olijhoek et al., 2018). They further stated that A:P ratio was higher in high CH_4_-emitting Jersey dairy cows compared to its counterpart Holstein dairy cows. However, in this study we only observed numerically higher proportion of propionate and lower proportion of acetate, and A:P ratio in the Holstein steers compared to the Jersey steers. The differences between the level of significance between these two studies may be due to the variation in the level of DMI and/or the production and absorption of individual VFAs by different breeds.

We hypothesized that Holstein steers would produce more CH_4_ (g/d) owing to their higher DMI, whereas Jersey steers would yield less CH_4_ (g/kg DMI) considering their greater feed efficiency (Spaans et al., 2018). However, our results showed numerically higher CH_4_ production (g/d), significantly higher CH_4_ yield (g/kg DMI), and a trend of higher CH_4_ intensity (g/kg BW^0.75^) in the Jersey steers than in the Holstein steers. This finding is in agreement with the results of Olijhoek et al. (2018) who reported that CH_4_ yield (g/kg DMI) was significantly higher in Jersey cows than in Holstein cows when fed high forage or high concentrate diets. Beside these, the recorded CH_4_ yield of Holstein steers in the present study was lower compared to some other studies; however, van Lingen et al. (2019) summarized the intercontinental databased to predict methane production (g/d) and yield (g/Kg DMI) by beef cattle where they presented that the range of methane production and yield with a high concentrate diets varies from 45 to 310 g/d, and 7.5 to 30.9 g/Kg DMI, respectively. Bharanidharan et al. (2018) reported that the methane yield of Holstein steers ranged from 10.3 to 11.3 g/kg DMI with a high concentrate TMR diet in Korea which was closer to our study. The pH of the above-mentioned study was varied from 6.3 to 6.7 which was also supported our study (pH ranges from 6.46 to 6.84). In addition, the methane yield varies remarkably between high and low methane emitting cattle. Danielsson et al. (2017) reported that yield of CH_4_ varies between low and high methane emiting cows (12.4 vs. 14.5) without affecting acetate and propionate proportion. Wallace et al. (2015) also reported that the CH_4_ yield varied between low and high emitting *Aberdeen Angus* (7.63 vs. 18.14) and Limousin cross (9.29 vs. 20.13) beef cattle with a high concentrate diet. *Methanobrevibacter* represents the most predominant methanogens and is responsible for most of the methanogenesis in ruminants (Leahy et al., 2013; Henderson et al., 2015). It had a positive association with higher CH_4_ emissions in cattle (Wallace et al., 2014, 2015). King et al. (2011) reported that *M. smithii, M. gottschalkii, M. millerae*, and *M. thaueri* of *Methanobrevibacter* (collectively referred to as the SGMT group) were more dominant in the Jersey breed while *M. ruminantium* and *M. olleyae* (collectively referred to as the RO group) were more abundant in the Holstein breed. The higher CH_4_ production and CH_4_ yield in the Jersey steers might be associated with the numerically higher relative abundance of *M. millerae* in the Jersey steers compared to the Holstein steers, which had a higher relative abundance of *M. olleyae*. Methane production also correlates with the VFAs production. The major methanogenesis substrates, formate and H_2_, are produced during acetate production, while propionate production competes with methanogens for H_2_ (Baldwin et al., 1963; Moss et al., 2000; Kittelmann et al., 2014; Islam and Lee, 2019). An earlier study also reported that the A:P ratio was proportional to the enteric CH_4_ production (Olijhoek et al., 2018). Although not-significant; however, numerically higher acetate proportion, and A:P ratio further correlate with the higher CH_4_ production in Jersey steer while numerically higher propionate proportion correlates with the lower CH_4_ production in the Holstein steers.

We further hypothesized that the above mentioned variation in VFAs and CH_4_ emissions might be linked with the variation in the rumen microbiota between these two breeds. The two breeds did not differ in the number of observed OTUs, Chao 1 richness estimate, Shannon, and Inverse Simpson diversity indexes, which is in contrast with the report of Paz et al. (2016) who reported significantly higher alpha diversity metrics, including Chao1 richness estimate and the number of observed OTUs in Holstein cows than in Jersey lactating cows. This discrepancy might be due to the variation of the microbiota affected by the host genetics and other factors, especially sex (Gonzalez-Recio et al., 2018; Li et al., 2019a) and physiological state (Bainbridge et al., 2016). Among the diverse populations of the rumen microbiota, bacteria are the most abundant domain, accounting for approximately 95% of the entire rumen prokaryotic microbiota (Zhou et al., 2015). Similarly, the present study found that the rumen microbiota was dominated by bacteria (>99%) regardless of breed or sampling hour. *Bacteroidetes* and *Firmicutes* were the most predominant bacterial phyla in the rumen of both the Holstein and Jersey steers regardless of the sampling hour, and these two phyla had similar relative abundance between the two breeds. This is consistent with the findings of several other studies (Jami and Mizrahi, 2012; Petri et al., 2013; Kim and Yu, 2014; Bharanidharan et al., 2018; Difford et al., 2018; Xue et al., 2018). However, the minor phyla, viz. *Fibrobacteres* and Candidatus *Melainabacteria*, varied between the Holstein and the Jersey steers, which might be due to breed effects. Furthermore, the PCoA plot and PCoA ellipses corresponding to the overall rumen microbiota of the two breeds were separated, indicating distinct different rumen microbiota harbored by each breed even fed the same TMR. Consistent with other studies (Bharanidharan et al., 2018; Difford et al., 2018; Xue et al., 2018), *Prevotella* had the most predominance in both breeds and at both sampling hours. Likewise, *Pr. ruminicola* was the most abundant *Prevotella* species, which did not differ between the two breeds. However, their higher abundance at 6 h after feeding indicates their dominancy increased over time. Sampling hours had little influence on the rumen bacterial community, but breeds had a significant influence on it. In this study, the Holstein steers had a significantly higher relative abundance of the genera *Ethanoligenens*, *Succinivibrio*, and the species *E. harbinense, S. dextrinosolvens, Pr. micans, Pr. copri, Pr. oris, Pr. baroniae*, and *T. succinifaciens* compared to Jersey steers, which indicates that these bacteria fit better to the rumen of the Holstein steers compared to the Jersey steers. Likewise, the Jersey steers had a significantly higher relative abundance of the genera *Capnocytophaga, Lachnoclostridium, Barnesiella, Oscillibacter*, *Galbibacter*, and the species *Ca. cynodegmi, G. mesophilus, B. intestinihominis, Pr. Shahii*, and *O. ruminantium* than the Holstein steers, indicating their higher activity in the rumen of Jersey steers. Usually, rumen microbial degradation of different feed particles produces VFAs and lactic acids which regulate the ruminal pH. Cattle with high forage diet, that leads to higher pH, had higher relative abundance of phyla *Firmicutes* and *Proteobacteria*, genera *Fibrobacter, Succinimonas, Polyplastron, Ostracodinium*, unclassified *Lachnospiraceae, Oribacterium, Pseudobutyrivibrio* while higher relative abundance of phylum *Bacteroidetes*, and genus *Prevotella* were observed in Cattle received high concentrate diet, which associated with lower pH (Zhang et al., 2017; Kim et al., 2018; Lee et al., 2019; Zeng et al., 2019; Wang et al., 2020). However, we fed the same TMR which minimizes the influence of diet types on pH and VFAs in our study. Furthermore, the pH and VFAs difference observed in the present study might be attributed to the variation of rumen microbiota between breeds through microbial niche modification in the rumen. This is because the ruminal pH and available metabolites influence initial niche modification (shaping of microbiota composition) by the microbes in the rumen ecosystem after feeding (Shaani et al., 2018). Therefore, rumen microbiota variation between the Holstein and the Jersey steers might be attributed to the influence of breeds, which influences their rumen fermentation and enteric CH_4_ emission between these two breeds. These results suggested that both the Holstein and the Jersey steers harbor a distinct dominant group of rumen microbes along with common microbes. The rumen microbiota variation between these two breeds was supported by Beecher et al. (2014) and Li et al. (2019a) who reported differences in the rumen microbiome among different breeds.

## Conclusion

Though, the Holstein and Jersey steers had similar species richness, Shannon, and inverse Simpson diversity indexes; however, the overall rumen microbiota were different between breeds, and both breeds have a distinct group of dominating rumen microbes. The higher enteric methane emissions was observed in the Jersey steers than that of Holstein steers even they received the same TMR diet. *M. millerae* was more abundant in the Jersey steers while *M. olleyae* in Holstein steers. The results of this study suggest that selective manipulation of rumen microbiome is needed to improve rumen fermentation and reduce CH_4_ emissions from steers of these breeds. To the best of our knowledge, this is the first comparative study investigating the rumen microbiota and enteric CH_4_ emissions on Holstein and Jersey steers when fed the same TMR diet.

## Data Availability Statement

The datasets generated for this study can be found in online repositories. The name of the repository (NCBI) and accession number (PRJNA660615) can be found in the following link: https://www.ncbi.nlm.nih.gov/sra/PRJNA660615.

## Ethics Statement

The animal study was reviewed and approved by Sunchon National University (SCNU) Institutional Animal Care and Use Committee (IACUC approval number: SCNU-IACUC-2020-06).

## Author Contributions

MI, S-HK, and Sa-SL designed and conceptualized the experiments. MI, S-HK, and SR performed feeding and management of steers. MI, S-HK, and A-RS performed the operation of GreenFeed. MI, S-HK, SR, and A-RS performed measurement of body weight and collection of rumen fluids. MI performed laboratory tests and statistical analysis. MI, S-HK, LM, ZY, Su-SL, Y-IC, and Sa-SL were performed data checking. MI wrote the first draft of the manuscript including tables and figures, which was revised by S-HK, LM, ZY, Su-SL, Y-IC, and Sa-SL. All authors contributed to the final manuscript revision, read and approved the final manuscript.

## Conflict of Interest

The authors declare that the research was conducted in the absence of any commercial or financial relationships that could be construed as a potential conflict of interest.

## Footnotes

1 https://github.com/PacificBiosciences/rDnaTools

2 https://www.ncbi.nlm.nih.gov/refseq/targetedloci/16S_process/

## Supplementary Material

Supplementary Table 1 Relative abundance of rumen microbes of Holstein and Jersey Steers at different taxonomic levels.

Supplementary Table 2 Bacterial genera each with relative abundance ≥ 0.1% at least in one breed and at one sampling hour.
