# Supplementary material for: Holstein and Jersey Steers Differ in Rumen Microbiota and Enteric Methane Emissions Even Fed the Same Total Mixed Ration
Source: Front Microbiol. 2021 Mar 18;12:601061. doi: 10.3389/fmicb.2021.601061 (PMC8044996; doi:10.3389/fmicb.2021.601061)
Supplement: Supplementary Table 1 — Relative abundance of rumen microbes of Holstein and Jersey Steers at different taxonomic levels. [file Table_1.docx]

Supplementary Material

**Supplementary Table 1:** Relative abundance of rumen microbes of Holstein and Jersey steers at different taxonomic levels.

| **Taxa** | **Holstein** | | **Jersey** | | **SEM** | ***P*-value** | | |
| --- | --- | --- | --- | --- | --- | --- | --- | --- |
|  | **0 h** | **6 h** | **0 h** | **6 h** |  | **Breed** | **Hour** | **B×H** |
| **Kingdom** |  |  |  |  |  |  |  |  |
| Archaea | 0.21 | 0.15 | 0.22 | 0.14 | 0.048 | 0.989 | 0.244 | 0.898 |
| Bacteria | 99.79 | 99.85 | 99.78 | 99.86 | 0.048 | 0.989 | 0.244 | 0.898 |
| **Archaeal Genera** |  |  |  |  |  |  |  |  |
| *Methanobrevibacter* | 96.83 | 100.00 | 100.00 | 95.87 | 1.575 | 0.833 | 0.833 | 0.117 |
| *Methanosphaera* | 3.17 | 0.00 | 0.00 | 1.75 | 1.071 | 0.675 | 0.675 | 0.159 |
| **Archaeal Species** |  |  |  |  |  |  |  |  |
| *Methanobrevibacter millerae* | 46.30 | 53.51 | 59.55 | 61.19 | 6.857 | 0.152 | 0.532 | 0.692 |
| *Methanobrevibacter olleyae* | 33.61 | 46.04 | 30.82 | 43.79 | 5.364 | 0.666 | 0.040 | 0.963 |
| **Bacterial Phyla** |  |  |  |  |  |  |  |  |
| Bacteroidetes | 61.98 | 62.05 | 64.84 | 62.60 | 2.379 | 0.515 | 0.680 | 0.658 |
| Firmicutes | 33.94 | 34.52 | 32.17 | 34.76 | 2.360 | 0.774 | 0.554 | 0.705 |
| Tenericutes | 1.56 | 0.89 | 1.55 | 0.74 | 0.235 | 0.775 | 0.011 | 0.804 |
| Proteobacteria | 1.18 | 1.27 | 0.24 | 0.86 | 0.362 | 0.118 | 0.404 | 0.532 |
| Spirochaetes | 0.83 | 0.67 | 0.71 | 0.53 | 0.141 | 0.388 | 0.271 | 0.955 |
| Synergistetes | 0.26 | 0.38 | 0.26 | 0.16 | 0.083 | 0.239 | 0.908 | 0.224 |
| Fibrobacteres | 0.09 | 0.10 | 0.04 | 0.02 | 0.020 | 0.004 | 0.758 | 0.539 |
| Candidatus Melainabacteria | 0.03 | 0.02 | 0.10 | 0.16 | 0.019 | 0.001 | 0.280 | 0.160 |

SEM, Standard error of the mean
