# Supplementary material for: Holstein and Jersey Steers Differ in Rumen Microbiota and Enteric Methane Emissions Even Fed the Same Total Mixed Ration
Source: Front Microbiol. 2021 Mar 18;12:601061. doi: 10.3389/fmicb.2021.601061 (PMC8044996; doi:10.3389/fmicb.2021.601061)
Supplement: Supplementary Table 2 — Bacterial genera each with relative abundance ≥ 0.1% at least in one breed and at one sampling hour. [file Table_2.docx]

Supplementary Material

**Supplementary Table 2:** Bacterial genera each with relative abundance ≥0.1% at least in one breed and at one sampling hour.

| **Genus** | **Holstein** | | **Jersey** | | **SEM** | ***P*-value** | | |
| --- | --- | --- | --- | --- | --- | --- | --- | --- |
|  | **0h** | **6h** | **0h** | **6h** |  | **Breed** | **Hour** | **B×H** |
| *Prevotella* | 35.19 | 42.11 | 32.79 | 34.17 | 2.567 | 0.068 | 0.136 | 0.312 |
| *Paludibacter* | 6.44 | 3.14 | 4.50 | 2.40 | 0.927 | 0.212 | 0.017 | 0.572 |
| *Capnocytophaga* | 4.73 | 3.02 | 7.76 | 7.09 | 0.704 | <0.001 | 0.131 | 0.502 |
| *Ruminococcus* | 4.51 | 4.57 | 3.54 | 4.06 | 0.509 | 0.195 | 0.607 | 0.685 |
| *Paraprevotella* | 4.22 | 4.18 | 4.97 | 5.00 | 1.360 | 0.607 | 0.998 | 0.979 |
| *Ethanoligenens* | 4.01 | 3.99 | 1.68 | 3.03 | 0.529 | 0.010 | 0.264 | 0.249 |
| *Intestinimonas* | 3.00 | 2.99 | 2.95 | 2.77 | 0.528 | 0.798 | 0.859 | 0.873 |
| *Galbibacter* | 2.95 | 2.30 | 3.65 | 3.69 | 0.406 | 0.024 | 0.485 | 0.433 |
| *Succiniclasticum* | 2.78 | 2.53 | 3.47 | 4.04 | 0.719 | 0.152 | 0.838 | 0.585 |
| *Flintibacter* | 2.56 | 2.93 | 2.95 | 3.87 | 0.382 | 0.104 | 0.115 | 0.493 |
| *Bacteroides* | 2.46 | 1.55 | 2.26 | 1.39 | 0.390 | 0.672 | 0.049 | 0.961 |
| *Christensenella* | 1.73 | 2.57 | 1.63 | 1.75 | 0.406 | 0.291 | 0.274 | 0.410 |
| *Lachnoclostridium* | 1.60 | 1.37 | 2.17 | 2.23 | 0.265 | 0.015 | 0.770 | 0.596 |
| *Barnesiella* | 1.42 | 1.49 | 3.14 | 3.56 | 0.386 | <0.001 | 0.546 | 0.673 |
| *Anaeroplasma* | 1.08 | 0.53 | 1.22 | 0.49 | 0.207 | 0.848 | 0.013 | 0.710 |
| *Lentimicrobium* | 0.86 | 0.77 | 0.77 | 0.70 | 0.150 | 0.663 | 0.642 | 0.957 |
| *Treponema* | 0.81 | 0.66 | 0.67 | 0.49 | 0.134 | 0.274 | 0.260 | 0.912 |
| *Eubacterium* | 0.77 | 0.80 | 1.02 | 0.83 | 0.177 | 0.463 | 0.684 | 0.559 |
| *Anaerobacterium* | 0.76 | 0.88 | 0.88 | 0.85 | 0.177 | 0.813 | 0.792 | 0.691 |
| UCG_F_Ruminococcaceae | 0.75 | 0.64 | 0.65 | 0.38 | 0.098 | 0.088 | 0.088 | 0.428 |
| *Vallitalea* | 0.61 | 0.60 | 0.81 | 0.73 | 0.096 | 0.135 | 0.655 | 0.751 |
| *Succinivibrio* | 0.58 | 0.53 | 0.06 | 0.25 | 0.149 | 0.039 | 0.680 | 0.502 |
| *Syntrophococcus* | 0.48 | 0.49 | 0.23 | 0.47 | 0.145 | 0.403 | 0.439 | 0.464 |
| *Gilliamella* | 0.47 | 0.62 | 0.10 | 0.46 | 0.214 | 0.291 | 0.311 | 0.677 |
| *Parabacteroides* | 0.26 | 0.21 | 0.71 | 0.37 | 0.187 | 0.234 | 0.444 | 0.564 |
| *Oscillibacter* | 0.25 | 0.18 | 0.65 | 0.51 | 0.043 | <0.001 | 0.034 | 0.478 |
| *Muribaculum* | 0.17 | 0.15 | 0.10 | 0.11 | 0.024 | 0.038 | 0.762 | 0.568 |
| *Prevotellamassilia* | 0.15 | 0.20 | 0.08 | 0.04 | 0.052 | 0.132 | 0.898 | 0.539 |
| *Fibrobacter* | 0.09 | 0.10 | 0.04 | 0.02 | 0.020 | 0.004 | 0.758 | 0.539 |
| *Sphingobacterium* | 0.06 | 0.10 | 0.15 | 0.10 | 0.071 | 0.551 | 0.955 | 0.536 |
| *Vampirovibrio* | 0.03 | 0.02 | 0.10 | 0.16 | 0.019 | 0.487 | 0.907 | 0.562 |
| *Olivibacter* | 0.00 | 0.00 | 0.18 | 0.15 | 0.067 | 0.001 | 0.280 | 0.160 |

SEM, Standard error of the mean
